# 1-Acetyl-3-ferrocenyl-5-(2-nitro­phen­yl)-2-pyrazoline

**DOI:** 10.1107/S1600536808004236

**Published:** 2008-03-05

**Authors:** Günseli Turgut Cin, Seda Demirel, Nevzat Karadayı, Orhan Büyükgüngör

**Affiliations:** a Department of Chemistry, Faculty of Arts and Sciences, Akdeniz University, TR-07058 Antalya, Turkey; b Samsun Vocational School, Ondokuz Mayıs University, TR-55139 Samsun, Turkey; c Department of Physics, Faculty of Arts and Sciences, Ondokuz Mayıs University, TR-55139 Samsun, Turkey

## Abstract

In the title compound, [Fe(C_5_H_5_)(C_16_H_14_N_3_O_3_)], the pyrazoline ring and the substituted cyclo­penta­dienyl ring are nearly coplanar, with a dihedral angle of 8.17 (2)°, while the nitro-substituted benzene ring is twisted out of the pyrazoline ring plane by 70.76 (1)°. The mol­ecules in the crystal structure are held together by three inter­molecular C—H⋯O hydrogen bonds. There is also an intra­molecular C—H⋯N hydrogen bond. The H atoms of the methyl group are disordered equally over two positions.

## Related literature

For related literature, see: Amr *et al.* (2006 ▶); Biot *et al.* (2004 ▶); Cremer & Pople (1975 ▶); Fang *et al.* (2003 ▶); Fouda *et al.* (2007 ▶); Guirado *et al.* (2004 ▶); Jaouen *et al.* (2004 ▶); Karthikeyan *et al.* (2007 ▶); Küçükgüzel *et al.* (2000 ▶); Kudar *et al.* (2005 ▶); Özdemir *et al.* (2007 ▶); Shi *et al.* (2006*a* ▶,*b* ▶); Shi *et al.* (2006 ▶); Zora *et al.* (2006 ▶, 2008 ▶).
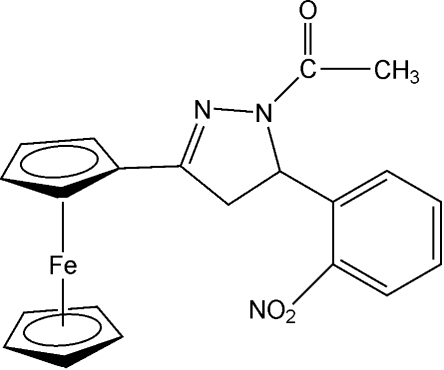


## Experimental

### 

#### Crystal data

[Fe(C_5_H_5_)(C_16_H_14_N_3_O_3_)]
*M* *_r_* = 417.24
Orthorhombic, 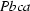

*a* = 8.6691 (6) Å
*b* = 13.4779 (7) Å
*c* = 31.4930 (15) Å
*V* = 3679.7 (4) Å^3^
*Z* = 8
Mo *K*α radiation
μ = 0.85 mm^−1^
*T* = 296 K
0.50 × 0.31 × 0.06 mm

#### Data collection

Stoe IPDSII diffractometer
Absorption correction: integration (*X-RED32*; Stoe & Cie, 2002 ▶) *T*_min_ = 0.636, *T*_max_ = 0.862
17799 measured reflections
3613 independent reflections
2409 reflections with *I* > 2σ(*I*)
*R*_int_ = 0.061

#### Refinement

*R*[*F* ^2^ > 2σ(*F* ^2^)] = 0.037
*wR*(*F* ^2^) = 0.086
*S* = 0.94
3613 reflections
253 parameters
H-atom parameters constrained
Δρ_max_ = 0.19 e Å^−3^
Δρ_min_ = −0.29 e Å^−3^

### 

Data collection: *X-AREA* (Stoe & Cie, 2002 ▶); cell refinement: *X-AREA*; data reduction: *X-RED32* (Stoe & Cie, 2002 ▶); program(s) used to solve structure: *SHELXS97* (Sheldrick, 2008 ▶); program(s) used to refine structure: *SHELXL97* (Sheldrick, 2008 ▶); molecular graphics: *ORTEP-3* (Farrugia, 1997 ▶); software used to prepare material for publication: *WinGX* (Farrugia, 1999 ▶) and *PLATON* (Spek, 2003 ▶).

## Supplementary Material

Crystal structure: contains datablocks global, I. DOI: 10.1107/S1600536808004236/hy2116sup1.cif

Structure factors: contains datablocks I. DOI: 10.1107/S1600536808004236/hy2116Isup2.hkl

Additional supplementary materials:  crystallographic information; 3D view; checkCIF report

## Figures and Tables

**Table 1:** Selected bond lengths (Å)

C12—Fe1	2.035 (3)
C13—Fe1	2.044 (3)
C14—Fe1	2.051 (3)
C15—Fe1	2.034 (3)
C16—Fe1	2.016 (3)
C17—Fe1	2.047 (3)
C18—Fe1	2.033 (3)
C19—Fe1	2.036 (3)
C20—Fe1	2.035 (3)
C21—Fe1	2.045 (3)

**Table 2:** Hydrogen-bond geometry (Å, °)

*D*—H⋯*A*	*D*—H	H⋯*A*	*D*⋯*A*	*D*—H⋯*A*
C11—H11*A*⋯N3	0.96	2.29	2.788 (4)	112
C8—H8*A*⋯O3^i^	0.97	2.57	3.476 (3)	154
C8—H8*B*⋯O2^ii^	0.97	2.63	3.551 (4)	158
C21—H21⋯O2^ii^	0.93	2.46	3.136 (4)	130

Symmetry codes: (i) 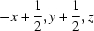; (ii) 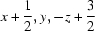.

**Fig. 1.**
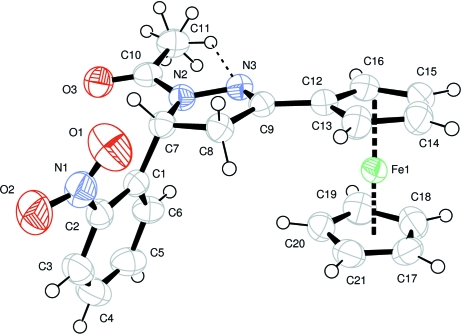
The molecular structure of the title compound. Displacement ellipsoids are drawn at the 50% probability level.

**Fig. 2.**
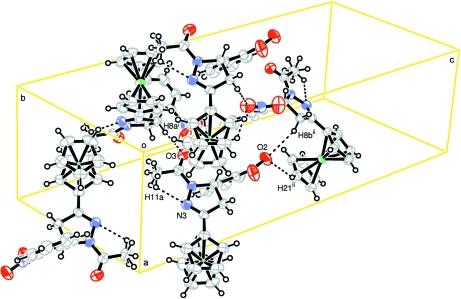
A view of the hydrogen-bonding network in the title compound. The dashed lines denote hydrogen bonds. [Symmetry codes: (i) -x + 1/2, y + 1/2, z; (ii) x + 1/2, y, -z + 3/2.]

## supplementary crystallographic information

### Comment

Pyrazolines are widely studied as five-membered heterocyclic compounds. Condensation of nitrogen-containing binucleophilic agents with α-β unsaturated ketones is one of the most suitable synthetic pathways for 2-pyrazolines (Kudar *et al.*, 2005), which possess widespread pharmaceutical properties such as antimicrobial (Küçükgüzel *et al.*, 2000), anticonvulsant (Karthikeyan *et al.*, 2007), antidepressant Özdemir *et al.*, 2007), antiandrogenic (Amr *et al.*, 2006), antifungal and anti-inflammatory (Guirado *et al.*, 2004) activities. Metallocenes are also known to exhibit a wide range of biological activity. Among them ferrocenyl compounds display interesting antibacterial (Fouda *et al.*, 2007), antitumor (Jaouen *et al.*, 2004), antimalarial and antifungal (Biot *et al.*, 2004) activities. Therefore, incorporation of a ferrocene fragment into a heterocyclic ring may enhance their biological activities or generate new medicinal properties (Fang *et al.*, 2003). As a part of an ongoing investigation of the chemistry of ferrocenyl pyrazolines, the title compound was synthesized and its crystal structure was determined.

The molecular structure of the title compound is shown in Fig. 1. The puckering parameters (Cremer & Pople, 1975) are q_2_=0.037 (3)Å and φ=43.3 (8)° for the pyrazoline ring, q_2_=0.008 (3)Å and φ=156.3 (2)° for the substituted cyclopentadienyl (Cp) ring and q_2_=0.004 (3)Å and φ=69.6 (5)° for the unsubstituted Cp ring. The dihedral angle of 8.17 (2)° between the pyrazoline ring and the substituted Cp ring indicates that they are conjugated with each other; this is in accord with the C9—C12 bond [1.456 (4) Å], showing a double-bond character (Shi *et al.*, 2006*a*). Furthermore, N3—C9 bond length [1.284 (3) Å] increases as a result of this conjugation. This observation is in good agreement with those reported for 2,6-bis(3-ferrocenyl-5-methyl-1*H*-pyrazol-1-ylcarbonyl)pyridine (Shi *et al.*, 2006*b*) and 1,3-bis(3-ferrocenyl-5-methyl-1*H*-pyrazol-1-ylcarbonyl)benzene (Shi *et al.*, 2006).

The Fe—*Cg*_s_ and Fe—*Cg*_as_ distances are 1.6398 (14) and 1.6544 (14) Å, respectively, and the *Cg*_s_—Fe—*Cg*_as_ angle is 177.78 (7)°, where *Cg*_s_ and *Cg*_as_ are the centroids of the substituted and unsubstituted Cp rings. The small dihedral angle of 3.68 (2)° between the unsubstituted and substituted Cp rings indicates that the two Cp rings are parallel to each other (Shi *et al.*, 2006*a*). The C12—*Cg*_s_—*Cg*_as_—C20 torsion angle of 3.71 (2)° indicates that the two Cp rings of the ferrocenyl group is nearly in an eclipsed conformation, as was previously observed in a ferrocene-containing compound (Zora *et al.*, 2008). The C—C bond distances in the Cp rings range from 1.389 (4) to 1.434 (4) Å, while Fe—C bond lengths range between 2.016 (3) and 2.051 (3)Å (Table 1), all of which are as expected (Zora *et al.*, 2006).

The pyrazoline ring and the nitro substituted phenyl group make a dihedral angle of 70.76 (1)°. The dihedral angle between the nitro plane and pheny ring is 18.30 (5)°. The dihedral angles between the phenyl ring and the substituted and unsubstituted Cp planes are 76.16 (1) and 76.12 (1)°, respectively. The molecules in crystal are held together by three intermolecular C—H···O hydrogen bonds (Table 2; Fig. 2). There is one intramolecular C—H···N hydrogen bond.

### Experimental

A mixture of 1-ferrocenyl-3-(2-nitrophenyl)-2-propenone (0.09 g, 0.24 mmol), 80% hydrazine hydrate (0.264 g, 5.28 mmol) and glacial acetic acid (10 ml) was refluxed under nitrogen atmosphere for 5 h. TLC indicated the formation of the reaction product. It was poured into ice-water to give dark orange solid. The participate was separated by filtration and washed with water. The solid product was dried at room temperature. Single crystals of the title compound suitable for X-ray measurements were obtained by recrystallization from methanol at room temperature (yield 50.5%; m.p. 425–426 K). IR (KBr, cm^-1^): 1653 (C?O), 1601 (C?C), 1574 (C?N), 1527 (*asym* N?O), 1333 (*sym* N?O), 1107 (C—N). ^1^H-NMR (CDCl_3_, p.p.m.): 2.33 (s, 1H, CH_3_), 2.89 (dd, 1H, pyrazoline H_4_), 3.84 (dd, 1H, pyrazoline H_4_), 4.04 (s, 5H, ferrocene), 4.36 (s, 2H, ferrocene), 4.53 (s, 1H, ferrocene), 4.60 (s, 1H, ferrocene), 6.03 (dd, 1H, pyrazoline H_5_), 7.23–8.05 (m, 4H, aromatic). ^13^C-NMR (CDCl_3_, p.p.m.): 21.75 (CH_3_), 43.83 (C4), 56.52 (C5), 67.30, 67.78 (C2', C5'), 69.46 (C6'–C10'), 70.61, 71.10 (C3', C4'), 74.86 (C1'), 125.56 (C8), 126.37 (C9), 128.50 (C11), 134.28 (C10), 137.33 (C6), 147.08 (C7), 156.63 (C3), 168.30 (C?O).

### Refinement

H atoms were positioned geometrically and refined as riding atoms, with C—H = 0.93 (aromatic), 0.98 (CH) and 0.97 Å (CH_2_) and with *U*_iso_(H) = 1.2*U*_eq_(C). H atoms of the methyl group (C11) show rotational disorder from a difference Fourier map. These H atoms were refined as riding atoms, with C—H = 0.96 Å and *U*_iso_(H) = 1.5*U*_eq_(C).

### Crystal data

**Table:**

[Fe(C_5_H_5_)(C_16_H_14_N_3_O_3_)]	*F*_000_ = 1728
*M**_r_* = 417.24	*D*_x_ = 1.506 Mg m^−^^3^
Orthorhombic, *P**b**c**a*	Mo *K*α radiation λ = 0.71073 Å
Hall symbol: -P 2ac 2ab	Cell parameters from 16565 reflections
*a* = 8.6691 (6) Å	θ = 1.6–27.9º
*b* = 13.4779 (7) Å	µ = 0.85 mm^−^^1^
*c* = 31.4930 (15) Å	*T* = 296 K
*V* = 3679.7 (4) Å^3^	Prism, red
*Z* = 8	0.50 × 0.31 × 0.06 mm

### Data collection

**Table:**

Stoe IPDSII diffractometer	3613 independent reflections
Radiation source: fine-focus sealed tube	2409 reflections with *I* > 2σ(*I*)
Monochromator: graphite	*R*_int_ = 0.061
*T* = 296(2) K	θ_max_ = 26.0º
ω scans	θ_min_ = 2.6º
Absorption correction: integration(X-RED32; Stoe & Cie, 2002)	*h* = −10→10
*T*_min_ = 0.636, *T*_max_ = 0.862	*k* = −16→13
17799 measured reflections	*l* = −38→38

### Refinement

**Table:**

Refinement on *F*^2^	Secondary atom site location: difference Fourier map
Least-squares matrix: full	Hydrogen site location: inferred from neighbouring sites
*R*[*F*^2^ > 2σ(*F*^2^)] = 0.037	H-atom parameters constrained
*wR*(*F*^2^) = 0.086	*w* = 1/[σ^2^(*F*_o_^2^) + (0.044*P*)^2^] where *P* = (*F*_o_^2^ + 2*F*_c_^2^)/3
*S* = 0.94	(Δ/σ)_max_ = 0.001
3613 reflections	Δρ_max_ = 0.19 e Å^−^^3^
253 parameters	Δρ_min_ = −0.29 e Å^−^^3^
Primary atom site location: structure-invariant direct methods	Extinction correction: none

### Special details

**Table:**

Geometry. All e.s.d.'s (except the e.s.d. in the dihedral angle between two l.s. planes) are estimated using the full covariance matrix. The cell e.s.d.'s are taken into account individually in the estimation of e.s.d.'s in distances, angles and torsion angles; correlations between e.s.d.'s in cell parameters are only used when they are defined by crystal symmetry. An approximate (isotropic) treatment of cell e.s.d.'s is used for estimating e.s.d.'s involving l.s. planes.

### Fractional atomic coordinates and isotropic or equivalent isotropic displacement parameters (Å^2^)

**Table:**

	*x*	*y*	*z*	*U*_iso_*/*U*_eq_	Occ. (<1)
C1	0.4226 (3)	0.55162 (19)	0.68996 (7)	0.0419 (6)	
C2	0.3958 (3)	0.5738 (2)	0.73262 (8)	0.0481 (7)	
C3	0.4637 (4)	0.5207 (2)	0.76547 (9)	0.0617 (8)	
H3	0.4434	0.5376	0.7935	0.074*	
C4	0.5601 (4)	0.4437 (2)	0.75645 (9)	0.0674 (9)	
H4	0.6058	0.4078	0.7783	0.081*	
C5	0.5895 (4)	0.4195 (2)	0.71485 (10)	0.0636 (8)	
H5	0.6561	0.3675	0.7085	0.076*	
C6	0.5201 (3)	0.4723 (2)	0.68231 (9)	0.0518 (7)	
H6	0.5398	0.4539	0.6544	0.062*	
C7	0.3550 (3)	0.61147 (18)	0.65354 (7)	0.0413 (6)	
H7	0.2483	0.6299	0.6601	0.050*	
C8	0.4494 (3)	0.70502 (18)	0.64348 (7)	0.0434 (6)	
H8A	0.3860	0.7642	0.6452	0.052*	
H8B	0.5363	0.7118	0.6627	0.052*	
C9	0.5024 (3)	0.68659 (19)	0.59887 (8)	0.0409 (6)	
C10	0.2617 (3)	0.48364 (19)	0.60260 (8)	0.0461 (6)	
C11	0.2627 (4)	0.4488 (2)	0.55716 (9)	0.0591 (8)	
H11A	0.3349	0.4877	0.5411	0.089*	0.50
H11B	0.1614	0.4562	0.5453	0.089*	0.50
H11C	0.2925	0.3802	0.5562	0.089*	0.50
H11D	0.1910	0.3950	0.5539	0.089*	0.50
H11E	0.3644	0.4265	0.5498	0.089*	0.50
H11F	0.2334	0.5025	0.5389	0.089*	0.50
C12	0.6069 (3)	0.7528 (2)	0.57636 (8)	0.0456 (6)	
C13	0.6708 (3)	0.84249 (19)	0.59343 (9)	0.0523 (7)	
H13	0.6467	0.8705	0.6196	0.063*	
C14	0.7769 (4)	0.8809 (2)	0.56346 (10)	0.0593 (8)	
H14	0.8346	0.9387	0.5665	0.071*	
C15	0.7799 (4)	0.8165 (3)	0.52835 (9)	0.0615 (8)	
H15	0.8397	0.8247	0.5041	0.074*	
C16	0.6772 (3)	0.7373 (2)	0.53603 (8)	0.0558 (7)	
H16	0.6586	0.6841	0.5179	0.067*	
C17	1.0530 (4)	0.7425 (2)	0.60978 (10)	0.0635 (8)	
H17	1.1130	0.7984	0.6149	0.076*	
C18	1.0552 (4)	0.6843 (3)	0.57284 (10)	0.0665 (9)	
H18	1.1166	0.6948	0.5491	0.080*	
C19	0.9461 (4)	0.6060 (2)	0.57837 (10)	0.0628 (8)	
H19	0.9238	0.5560	0.5590	0.075*	
C20	0.8795 (4)	0.6185 (2)	0.61815 (9)	0.0560 (8)	
H20	0.8039	0.5782	0.6300	0.067*	
C21	0.9452 (4)	0.7019 (2)	0.63740 (9)	0.0581 (8)	
H21	0.9208	0.7262	0.6642	0.070*	
N1	0.2950 (3)	0.6566 (2)	0.74518 (7)	0.0580 (7)	
N2	0.3604 (3)	0.55775 (15)	0.61266 (6)	0.0421 (5)	
N3	0.4499 (2)	0.60674 (15)	0.58194 (6)	0.0446 (5)	
O1	0.2640 (4)	0.72062 (19)	0.71980 (7)	0.0857 (8)	
O2	0.2464 (3)	0.6580 (2)	0.78155 (7)	0.0818 (7)	
O3	0.1803 (2)	0.44698 (15)	0.63000 (6)	0.0583 (5)	
Fe1	0.84006 (4)	0.74057 (2)	0.581679 (10)	0.04091 (12)	

### Atomic displacement parameters (Å^2^)

**Table:**

	*U*^11^	*U*^22^	*U*^33^	*U*^12^	*U*^13^	*U*^23^
C1	0.0399 (16)	0.0440 (15)	0.0416 (13)	−0.0072 (12)	−0.0011 (11)	0.0002 (10)
C2	0.0490 (17)	0.0539 (16)	0.0413 (13)	−0.0120 (13)	0.0013 (12)	−0.0019 (12)
C3	0.074 (2)	0.068 (2)	0.0428 (14)	−0.0177 (18)	−0.0069 (15)	0.0037 (14)
C4	0.082 (3)	0.062 (2)	0.0585 (19)	−0.0102 (18)	−0.0196 (17)	0.0154 (15)
C5	0.066 (2)	0.0507 (17)	0.074 (2)	0.0045 (15)	−0.0186 (17)	0.0042 (14)
C6	0.0549 (19)	0.0486 (16)	0.0519 (14)	0.0029 (13)	−0.0086 (13)	−0.0040 (12)
C7	0.0393 (15)	0.0431 (13)	0.0415 (12)	0.0030 (12)	0.0012 (11)	−0.0015 (10)
C8	0.0465 (17)	0.0377 (14)	0.0459 (13)	−0.0015 (11)	−0.0004 (12)	−0.0004 (10)
C9	0.0366 (15)	0.0453 (14)	0.0410 (12)	0.0036 (12)	0.0000 (11)	0.0027 (11)
C10	0.0453 (17)	0.0407 (15)	0.0523 (14)	0.0038 (12)	−0.0092 (14)	−0.0006 (12)
C11	0.065 (2)	0.0535 (17)	0.0591 (17)	−0.0014 (15)	−0.0124 (15)	−0.0099 (14)
C12	0.0397 (13)	0.0507 (15)	0.0463 (13)	0.0012 (12)	−0.0023 (10)	0.0069 (12)
C13	0.0491 (17)	0.0425 (14)	0.0654 (17)	0.0040 (13)	0.0066 (14)	0.0035 (12)
C14	0.0485 (18)	0.0491 (17)	0.080 (2)	0.0027 (14)	0.0036 (16)	0.0182 (15)
C15	0.0476 (18)	0.081 (2)	0.0553 (16)	−0.0004 (16)	0.0005 (14)	0.0281 (16)
C16	0.0494 (17)	0.0726 (19)	0.0453 (13)	−0.0041 (15)	−0.0064 (12)	0.0084 (13)
C17	0.0484 (18)	0.0617 (19)	0.080 (2)	−0.0082 (16)	−0.0178 (15)	0.0164 (17)
C18	0.051 (2)	0.077 (2)	0.072 (2)	0.0194 (16)	0.0159 (15)	0.0255 (17)
C19	0.066 (2)	0.0487 (17)	0.0741 (19)	0.0129 (14)	0.0016 (18)	−0.0015 (15)
C20	0.0488 (19)	0.0531 (17)	0.0661 (18)	0.0028 (13)	0.0005 (14)	0.0162 (14)
C21	0.063 (2)	0.0604 (19)	0.0511 (15)	0.0017 (15)	−0.0096 (15)	0.0077 (13)
N1	0.0575 (17)	0.0695 (17)	0.0470 (13)	−0.0097 (13)	0.0061 (12)	−0.0141 (12)
N2	0.0446 (14)	0.0448 (12)	0.0370 (10)	−0.0040 (10)	−0.0023 (9)	−0.0012 (8)
N3	0.0421 (13)	0.0491 (12)	0.0427 (10)	−0.0009 (10)	−0.0021 (11)	0.0011 (10)
O1	0.118 (2)	0.0697 (16)	0.0695 (14)	0.0264 (15)	0.0219 (14)	−0.0057 (12)
O2	0.0805 (18)	0.114 (2)	0.0514 (12)	−0.0059 (15)	0.0203 (12)	−0.0183 (12)
O3	0.0565 (14)	0.0540 (12)	0.0644 (12)	−0.0097 (10)	−0.0030 (10)	0.0044 (9)
Fe1	0.0390 (2)	0.0419 (2)	0.04183 (17)	0.00169 (17)	0.00009 (16)	0.00513 (16)

### Geometric parameters (Å, °)

**Table:**

C1—C6	1.384 (4)	C12—Fe1	2.035 (3)
C1—C2	1.396 (3)	C13—C14	1.416 (4)
C1—C7	1.519 (3)	C13—Fe1	2.044 (3)
C2—C3	1.389 (4)	C13—H13	0.9300
C2—N1	1.472 (4)	C14—C15	1.407 (4)
C3—C4	1.362 (5)	C14—Fe1	2.051 (3)
C3—H3	0.9300	C14—H14	0.9300
C4—C5	1.374 (4)	C15—C16	1.411 (4)
C4—H4	0.9300	C15—Fe1	2.034 (3)
C5—C6	1.385 (4)	C15—H15	0.9300
C5—H5	0.9300	C16—Fe1	2.016 (3)
C6—H6	0.9300	C16—H16	0.9300
C7—N2	1.478 (3)	C17—C21	1.389 (4)
C7—C8	1.536 (3)	C17—C18	1.403 (5)
C7—H7	0.9800	C17—Fe1	2.047 (3)
C8—C9	1.499 (3)	C17—H17	0.9300
C8—H8A	0.9700	C18—C19	1.428 (5)
C8—H8B	0.9700	C18—Fe1	2.033 (3)
C9—N3	1.284 (3)	C18—H18	0.9300
C9—C12	1.456 (4)	C19—C20	1.390 (4)
C10—O3	1.219 (3)	C19—Fe1	2.036 (3)
C10—N2	1.353 (3)	C19—H19	0.9300
C10—C11	1.506 (4)	C20—C21	1.398 (4)
C11—H11A	0.9600	C20—Fe1	2.035 (3)
C11—H11B	0.9600	C20—H20	0.9300
C11—H11C	0.9600	C21—Fe1	2.045 (3)
C11—H11D	0.9600	C21—H21	0.9300
C11—H11E	0.9600	N1—O1	1.206 (3)
C11—H11F	0.9600	N1—O2	1.221 (3)
C12—C16	1.424 (4)	N2—N3	1.405 (3)
C12—C13	1.434 (4)		
			
C6—C1—C2	115.7 (2)	C12—C16—H16	125.9
C6—C1—C7	120.9 (2)	Fe1—C16—H16	125.2
C2—C1—C7	123.3 (2)	C21—C17—C18	108.0 (3)
C3—C2—C1	122.4 (3)	C21—C17—Fe1	70.08 (17)
C3—C2—N1	116.3 (3)	C18—C17—Fe1	69.33 (18)
C1—C2—N1	121.3 (2)	C21—C17—H17	126.0
C4—C3—C2	119.8 (3)	C18—C17—H17	126.0
C4—C3—H3	120.1	Fe1—C17—H17	126.1
C2—C3—H3	120.1	C17—C18—C19	107.6 (3)
C3—C4—C5	119.6 (3)	C17—C18—Fe1	70.44 (18)
C3—C4—H4	120.2	C19—C18—Fe1	69.60 (18)
C5—C4—H4	120.2	C17—C18—H18	126.2
C4—C5—C6	120.2 (3)	C19—C18—H18	126.2
C4—C5—H5	119.9	Fe1—C18—H18	125.4
C6—C5—H5	119.9	C20—C19—C18	107.2 (3)
C1—C6—C5	122.2 (3)	C20—C19—Fe1	70.00 (17)
C1—C6—H6	118.9	C18—C19—Fe1	69.32 (17)
C5—C6—H6	118.9	C20—C19—H19	126.4
N2—C7—C1	112.7 (2)	C18—C19—H19	126.4
N2—C7—C8	101.87 (19)	Fe1—C19—H19	125.8
C1—C7—C8	112.7 (2)	C19—C20—C21	108.6 (3)
N2—C7—H7	109.8	C19—C20—Fe1	70.08 (16)
C1—C7—H7	109.8	C21—C20—Fe1	70.33 (16)
C8—C7—H7	109.8	C19—C20—H20	125.7
C9—C8—C7	102.7 (2)	C21—C20—H20	125.7
C9—C8—H8A	111.2	Fe1—C20—H20	125.5
C7—C8—H8A	111.2	C17—C21—C20	108.6 (3)
C9—C8—H8B	111.2	C17—C21—Fe1	70.24 (16)
C7—C8—H8B	111.2	C20—C21—Fe1	69.58 (16)
H8A—C8—H8B	109.1	C17—C21—H21	125.7
N3—C9—C12	122.1 (2)	C20—C21—H21	125.7
N3—C9—C8	114.8 (2)	Fe1—C21—H21	126.1
C12—C9—C8	123.1 (2)	O1—N1—O2	122.3 (3)
O3—C10—N2	120.0 (2)	O1—N1—C2	119.8 (2)
O3—C10—C11	123.3 (3)	O2—N1—C2	117.9 (3)
N2—C10—C11	116.7 (3)	C10—N2—N3	122.3 (2)
C10—C11—H11A	109.5	C10—N2—C7	123.1 (2)
C10—C11—H11B	109.5	N3—N2—C7	112.77 (19)
H11A—C11—H11B	109.5	C9—N3—N2	107.67 (19)
C10—C11—H11C	109.5	C16—Fe1—C18	122.47 (13)
H11A—C11—H11C	109.5	C16—Fe1—C15	40.78 (12)
H11B—C11—H11C	109.5	C18—Fe1—C15	108.04 (13)
C10—C11—H11D	109.5	C16—Fe1—C12	41.17 (11)
H11A—C11—H11D	141.1	C18—Fe1—C12	158.50 (14)
H11B—C11—H11D	56.3	C15—Fe1—C12	68.71 (11)
H11C—C11—H11D	56.3	C16—Fe1—C20	120.16 (13)
C10—C11—H11E	109.5	C18—Fe1—C20	67.76 (12)
H11A—C11—H11E	56.3	C15—Fe1—C20	156.25 (14)
H11B—C11—H11E	141.1	C12—Fe1—C20	106.19 (11)
H11C—C11—H11E	56.3	C16—Fe1—C19	105.08 (13)
H11D—C11—H11E	109.5	C18—Fe1—C19	41.08 (13)
C10—C11—H11F	109.5	C15—Fe1—C19	121.42 (14)
H11A—C11—H11F	56.3	C12—Fe1—C19	121.10 (13)
H11B—C11—H11F	56.3	C20—Fe1—C19	39.92 (12)
H11C—C11—H11F	141.1	C16—Fe1—C13	68.97 (12)
H11D—C11—H11F	109.5	C18—Fe1—C13	159.13 (14)
H11E—C11—H11F	109.5	C15—Fe1—C13	68.14 (12)
C16—C12—C13	107.0 (2)	C12—Fe1—C13	41.18 (11)
C16—C12—C9	127.6 (3)	C20—Fe1—C13	124.17 (12)
C13—C12—C9	125.1 (2)	C19—Fe1—C13	158.78 (13)
C16—C12—Fe1	68.67 (15)	C16—Fe1—C21	156.74 (12)
C13—C12—Fe1	69.73 (16)	C18—Fe1—C21	67.26 (13)
C9—C12—Fe1	121.89 (18)	C15—Fe1—C21	161.94 (14)
C14—C13—C12	108.0 (3)	C12—Fe1—C21	122.28 (12)
C14—C13—Fe1	70.04 (17)	C20—Fe1—C21	40.08 (12)
C12—C13—Fe1	69.09 (15)	C19—Fe1—C21	67.40 (13)
C14—C13—H13	126.0	C13—Fe1—C21	109.62 (12)
C12—C13—H13	126.0	C16—Fe1—C17	160.09 (13)
Fe1—C13—H13	126.5	C18—Fe1—C17	40.24 (13)
C15—C14—C13	108.1 (3)	C15—Fe1—C17	125.55 (13)
C15—C14—Fe1	69.23 (17)	C12—Fe1—C17	158.46 (12)
C13—C14—Fe1	69.50 (16)	C20—Fe1—C17	67.35 (12)
C15—C14—H14	126.0	C19—Fe1—C17	68.06 (14)
C13—C14—H14	126.0	C13—Fe1—C17	124.09 (13)
Fe1—C14—H14	126.9	C21—Fe1—C17	39.68 (12)
C14—C15—C16	108.7 (3)	C16—Fe1—C14	68.53 (13)
C14—C15—Fe1	70.50 (16)	C18—Fe1—C14	123.41 (13)
C16—C15—Fe1	68.89 (15)	C15—Fe1—C14	40.27 (13)
C14—C15—H15	125.6	C12—Fe1—C14	68.73 (12)
C16—C15—H15	125.6	C20—Fe1—C14	161.46 (12)
Fe1—C15—H15	126.5	C19—Fe1—C14	158.08 (13)
C15—C16—C12	108.2 (3)	C13—Fe1—C14	40.47 (11)
C15—C16—Fe1	70.33 (16)	C21—Fe1—C14	126.43 (13)
C12—C16—Fe1	70.16 (15)	C17—Fe1—C14	110.47 (13)
C15—C16—H16	125.9		
			
C6—C1—C2—C3	0.7 (4)	C16—C15—Fe1—C14	−120.1 (3)
C7—C1—C2—C3	−177.3 (3)	C13—C12—Fe1—C16	−118.6 (2)
C6—C1—C2—N1	179.6 (3)	C9—C12—Fe1—C16	121.9 (3)
C7—C1—C2—N1	1.7 (4)	C16—C12—Fe1—C18	−47.3 (4)
C1—C2—C3—C4	−0.1 (5)	C13—C12—Fe1—C18	−166.0 (3)
N1—C2—C3—C4	−179.1 (3)	C9—C12—Fe1—C18	74.6 (4)
C2—C3—C4—C5	0.1 (5)	C16—C12—Fe1—C15	37.92 (18)
C3—C4—C5—C6	−0.7 (5)	C13—C12—Fe1—C15	−80.71 (18)
C2—C1—C6—C5	−1.3 (4)	C9—C12—Fe1—C15	159.9 (3)
C7—C1—C6—C5	176.7 (3)	C16—C12—Fe1—C20	−117.56 (18)
C4—C5—C6—C1	1.3 (5)	C13—C12—Fe1—C20	123.81 (17)
C6—C1—C7—N2	19.3 (3)	C9—C12—Fe1—C20	4.4 (2)
C2—C1—C7—N2	−162.9 (2)	C16—C12—Fe1—C19	−76.8 (2)
C6—C1—C7—C8	−95.4 (3)	C13—C12—Fe1—C19	164.57 (17)
C2—C1—C7—C8	82.5 (3)	C9—C12—Fe1—C19	45.1 (3)
N2—C7—C8—C9	−3.4 (3)	C16—C12—Fe1—C13	118.6 (2)
C1—C7—C8—C9	117.6 (2)	C9—C12—Fe1—C13	−119.4 (3)
C7—C8—C9—N3	3.9 (3)	C16—C12—Fe1—C21	−158.19 (18)
C7—C8—C9—C12	−175.3 (2)	C13—C12—Fe1—C21	83.18 (18)
N3—C9—C12—C16	−6.5 (4)	C9—C12—Fe1—C21	−36.3 (3)
C8—C9—C12—C16	172.7 (3)	C16—C12—Fe1—C17	173.3 (3)
N3—C9—C12—C13	−179.4 (3)	C13—C12—Fe1—C17	54.7 (4)
C8—C9—C12—C13	−0.3 (4)	C9—C12—Fe1—C17	−64.7 (4)
N3—C9—C12—Fe1	−93.0 (3)	C16—C12—Fe1—C14	81.28 (19)
C8—C9—C12—Fe1	86.2 (3)	C13—C12—Fe1—C14	−37.35 (16)
C16—C12—C13—C14	0.7 (3)	C9—C12—Fe1—C14	−156.8 (2)
C9—C12—C13—C14	174.8 (3)	C19—C20—Fe1—C16	77.0 (2)
Fe1—C12—C13—C14	59.4 (2)	C21—C20—Fe1—C16	−163.69 (18)
C16—C12—C13—Fe1	−58.77 (19)	C19—C20—Fe1—C18	−38.7 (2)
C9—C12—C13—Fe1	115.4 (3)	C21—C20—Fe1—C18	80.6 (2)
C12—C13—C14—C15	−0.2 (3)	C19—C20—Fe1—C15	45.6 (4)
Fe1—C13—C14—C15	58.6 (2)	C21—C20—Fe1—C15	165.0 (3)
C12—C13—C14—Fe1	−58.83 (19)	C19—C20—Fe1—C12	119.41 (19)
C13—C14—C15—C16	−0.4 (3)	C21—C20—Fe1—C12	−121.23 (19)
Fe1—C14—C15—C16	58.4 (2)	C21—C20—Fe1—C19	119.4 (3)
C13—C14—C15—Fe1	−58.8 (2)	C19—C20—Fe1—C13	160.80 (19)
C14—C15—C16—C12	0.8 (3)	C21—C20—Fe1—C13	−79.8 (2)
Fe1—C15—C16—C12	60.21 (19)	C19—C20—Fe1—C21	−119.4 (3)
C14—C15—C16—Fe1	−59.4 (2)	C19—C20—Fe1—C17	−82.4 (2)
C13—C12—C16—C15	−0.9 (3)	C21—C20—Fe1—C17	36.95 (19)
C9—C12—C16—C15	−174.8 (3)	C19—C20—Fe1—C14	−169.4 (4)
Fe1—C12—C16—C15	−60.3 (2)	C21—C20—Fe1—C14	−50.0 (5)
C13—C12—C16—Fe1	59.44 (19)	C20—C19—Fe1—C16	−119.27 (19)
C9—C12—C16—Fe1	−114.5 (3)	C18—C19—Fe1—C16	122.48 (19)
C21—C17—C18—C19	−0.3 (4)	C20—C19—Fe1—C18	118.2 (3)
Fe1—C17—C18—C19	−59.9 (2)	C20—C19—Fe1—C15	−160.29 (19)
C21—C17—C18—Fe1	59.7 (2)	C18—C19—Fe1—C15	81.5 (2)
C17—C18—C19—C20	0.4 (3)	C20—C19—Fe1—C12	−77.7 (2)
Fe1—C18—C19—C20	−60.0 (2)	C18—C19—Fe1—C12	164.07 (18)
C17—C18—C19—Fe1	60.4 (2)	C18—C19—Fe1—C20	−118.2 (3)
C18—C19—C20—C21	−0.4 (3)	C20—C19—Fe1—C13	−48.7 (4)
Fe1—C19—C20—C21	−60.0 (2)	C18—C19—Fe1—C13	−167.0 (3)
C18—C19—C20—Fe1	59.6 (2)	C20—C19—Fe1—C21	37.44 (19)
C18—C17—C21—C20	0.0 (4)	C18—C19—Fe1—C21	−80.8 (2)
Fe1—C17—C21—C20	59.2 (2)	C20—C19—Fe1—C17	80.5 (2)
C18—C17—C21—Fe1	−59.2 (2)	C18—C19—Fe1—C17	−37.78 (19)
C19—C20—C21—C17	0.2 (3)	C20—C19—Fe1—C14	171.0 (3)
Fe1—C20—C21—C17	−59.6 (2)	C18—C19—Fe1—C14	52.7 (4)
C19—C20—C21—Fe1	59.9 (2)	C14—C13—Fe1—C16	−81.2 (2)
C3—C2—N1—O1	161.2 (3)	C12—C13—Fe1—C16	38.25 (15)
C1—C2—N1—O1	−17.8 (4)	C14—C13—Fe1—C18	46.1 (4)
C3—C2—N1—O2	−18.3 (4)	C12—C13—Fe1—C18	165.5 (3)
C1—C2—N1—O2	162.7 (3)	C14—C13—Fe1—C15	−37.22 (19)
O3—C10—N2—N3	−176.0 (2)	C12—C13—Fe1—C15	82.21 (17)
C11—C10—N2—N3	5.6 (4)	C14—C13—Fe1—C12	−119.4 (2)
O3—C10—N2—C7	−12.4 (4)	C14—C13—Fe1—C20	165.90 (19)
C11—C10—N2—C7	169.3 (2)	C12—C13—Fe1—C20	−74.68 (19)
C1—C7—N2—C10	76.4 (3)	C14—C13—Fe1—C19	−158.4 (3)
C8—C7—N2—C10	−162.6 (2)	C12—C13—Fe1—C19	−39.0 (4)
C1—C7—N2—N3	−118.6 (2)	C14—C13—Fe1—C21	123.6 (2)
C8—C7—N2—N3	2.4 (3)	C12—C13—Fe1—C21	−116.97 (16)
C12—C9—N3—N2	176.7 (2)	C14—C13—Fe1—C17	81.8 (2)
C8—C9—N3—N2	−2.5 (3)	C12—C13—Fe1—C17	−158.78 (15)
C10—N2—N3—C9	165.0 (2)	C12—C13—Fe1—C14	119.4 (2)
C7—N2—N3—C9	−0.1 (3)	C17—C21—Fe1—C16	157.6 (3)
C15—C16—Fe1—C18	−79.9 (2)	C20—C21—Fe1—C16	37.9 (4)
C12—C16—Fe1—C18	161.37 (17)	C17—C21—Fe1—C18	37.7 (2)
C12—C16—Fe1—C15	−118.8 (3)	C20—C21—Fe1—C18	−82.0 (2)
C15—C16—Fe1—C12	118.8 (3)	C17—C21—Fe1—C15	−40.6 (5)
C15—C16—Fe1—C20	−161.29 (19)	C20—C21—Fe1—C15	−160.3 (4)
C12—C16—Fe1—C20	80.0 (2)	C17—C21—Fe1—C12	−164.09 (19)
C15—C16—Fe1—C19	−120.9 (2)	C20—C21—Fe1—C12	76.2 (2)
C12—C16—Fe1—C19	120.30 (18)	C17—C21—Fe1—C20	119.7 (3)
C15—C16—Fe1—C13	80.5 (2)	C17—C21—Fe1—C19	82.4 (2)
C12—C16—Fe1—C13	−38.25 (16)	C20—C21—Fe1—C19	−37.29 (18)
C15—C16—Fe1—C21	171.5 (3)	C17—C21—Fe1—C13	−120.1 (2)
C12—C16—Fe1—C21	52.7 (4)	C20—C21—Fe1—C13	120.17 (19)
C15—C16—Fe1—C17	−54.1 (5)	C20—C21—Fe1—C17	−119.7 (3)
C12—C16—Fe1—C17	−172.8 (3)	C17—C21—Fe1—C14	−77.9 (2)
C15—C16—Fe1—C14	36.94 (19)	C20—C21—Fe1—C14	162.37 (18)
C12—C16—Fe1—C14	−81.82 (18)	C21—C17—Fe1—C16	−153.8 (3)
C17—C18—Fe1—C16	166.73 (17)	C18—C17—Fe1—C16	−34.7 (5)
C19—C18—Fe1—C16	−74.9 (2)	C21—C17—Fe1—C18	−119.2 (3)
C17—C18—Fe1—C15	124.18 (19)	C21—C17—Fe1—C15	165.6 (2)
C19—C18—Fe1—C15	−117.4 (2)	C18—C17—Fe1—C15	−75.2 (2)
C17—C18—Fe1—C12	−158.3 (3)	C21—C17—Fe1—C12	39.1 (4)
C19—C18—Fe1—C12	−39.9 (4)	C18—C17—Fe1—C12	158.3 (3)
C17—C18—Fe1—C20	−80.74 (19)	C21—C17—Fe1—C20	−37.31 (18)
C19—C18—Fe1—C20	37.64 (18)	C18—C17—Fe1—C20	81.9 (2)
C17—C18—Fe1—C19	−118.4 (3)	C21—C17—Fe1—C19	−80.6 (2)
C17—C18—Fe1—C13	48.4 (4)	C18—C17—Fe1—C19	38.56 (18)
C19—C18—Fe1—C13	166.8 (3)	C21—C17—Fe1—C13	79.6 (2)
C17—C18—Fe1—C21	−37.20 (18)	C18—C17—Fe1—C13	−161.24 (18)
C19—C18—Fe1—C21	81.2 (2)	C18—C17—Fe1—C21	119.2 (3)
C19—C18—Fe1—C17	118.4 (3)	C21—C17—Fe1—C14	122.9 (2)
C17—C18—Fe1—C14	82.5 (2)	C18—C17—Fe1—C14	−118.0 (2)
C19—C18—Fe1—C14	−159.15 (19)	C15—C14—Fe1—C16	−37.39 (18)
C14—C15—Fe1—C16	120.1 (3)	C13—C14—Fe1—C16	82.35 (19)
C14—C15—Fe1—C18	−120.8 (2)	C15—C14—Fe1—C18	78.2 (2)
C16—C15—Fe1—C18	119.1 (2)	C13—C14—Fe1—C18	−162.08 (19)
C14—C15—Fe1—C12	81.82 (19)	C13—C14—Fe1—C15	119.7 (3)
C16—C15—Fe1—C12	−38.28 (18)	C15—C14—Fe1—C12	−81.75 (19)
C14—C15—Fe1—C20	163.6 (3)	C13—C14—Fe1—C12	37.98 (17)
C16—C15—Fe1—C20	43.5 (4)	C15—C14—Fe1—C20	−159.1 (4)
C14—C15—Fe1—C19	−163.88 (18)	C13—C14—Fe1—C20	−39.3 (5)
C16—C15—Fe1—C19	76.0 (2)	C15—C14—Fe1—C19	39.4 (4)
C14—C15—Fe1—C13	37.39 (18)	C13—C14—Fe1—C19	159.1 (3)
C16—C15—Fe1—C13	−82.7 (2)	C15—C14—Fe1—C13	−119.7 (3)
C14—C15—Fe1—C21	−49.0 (5)	C15—C14—Fe1—C21	163.10 (19)
C16—C15—Fe1—C21	−169.1 (4)	C13—C14—Fe1—C21	−77.2 (2)
C14—C15—Fe1—C17	−79.7 (2)	C15—C14—Fe1—C17	121.3 (2)
C16—C15—Fe1—C17	160.19 (19)	C13—C14—Fe1—C17	−118.96 (19)

### Hydrogen-bond geometry (Å, °)

**Table:**

*D*—H···*A*	*D*—H	H···*A*	*D*···*A*	*D*—H···*A*
C11—H11A···N3	0.96	2.29	2.788 (4)	112
C8—H8A···O3^i^	0.97	2.57	3.476 (3)	154
C8—H8B···O2^ii^	0.97	2.63	3.551 (4)	158
C21—H21···O2^ii^	0.93	2.46	3.136 (4)	130

Symmetry codes: (i) −*x*+1/2, *y*+1/2, *z*; (ii) *x*+1/2, *y*, −*z*+3/2.
